# Reporting of hypoglycaemia in clinical trials of basal insulins: A need for consensus

**DOI:** 10.1111/dom.13732

**Published:** 2019-04-29

**Authors:** Brian M. Frier, Alexandria Ratzki‐Leewing, Stewart B. Harris

**Affiliations:** ^1^ British Heart Foundation Centre for Cardiovascular Science The Queen's Medical Research Institute, University of Edinburgh Edinburgh UK; ^2^ Department of Epidemiology and Biostatistics Schulich School of Medicine and Dentistry, Western University London Ontario, Canada; ^3^ Department of Family Medicine Schulich School of Medicine and Dentistry, Western University London Ontario, Canada

**Keywords:** basal insulin, insulin therapy, hypoglycaemia

## Abstract

Hypoglycaemia is a common side‐effect of diabetes therapies, particularly insulin, and imposes a substantial burden on individuals and healthcare systems. Consequently, regulatory approval of newer basal insulin (BI) therapies has relied on demonstration of a balance between achievement of good glycaemic control and less hypoglycaemia. Randomized controlled trials (RCTs) are the gold standard for assessing efficacy and safety, including hypoglycaemia risk, of BIs and are invaluable for obtaining regulatory approval. However, their highly selected patient populations and their conditions lead to results that may not be representative of real‐life situations. Real‐world evidence (RWE) studies are more representative of clinical practice, but they also have limitations. As such, data both from RCTs and RWE studies provide a fuller picture of the hypoglycaemia risk with BI therapies. However, substantial differences exist in the way hypoglycaemia is reported across these studies, which confounds comparisons of hypoglycaemia frequency among different BIs. This problem is ongoing and persists in recent trials of second‐generation BI analogues. Although they provide a lower risk of hypoglycaemia when compared with earlier BIs, they do not eliminate it. This review describes differences in the way hypoglycaemia is reported across RCTs and RWE studies of second‐generation BI analogues and examines potential reasons for these differences. For studies of BIs, there is a need to standardize aspects of design, analysis and methods of reporting to better enable interpretation of the efficacy and safety of such insulins among studies; such aspects include length of follow‐up, glycaemic targets, hypoglycaemia definitions and time intervals for determining nocturnal events.

## 1 INTRODUCTION

Hypoglycaemia remains a common side‐effect of diabetes treatment with insulin and is associated with a range of morbidities, including falls and accidents, and adverse cardiovascular events.^1^ Severe hypoglycaemia, for which the individual requires external assistance (aid from another person to actively administer carbohydrate or parenteral therapy), is also associated with seizures, coma and increased risk of mortality.^1^

Almost every aspect of daily life can be influenced by hypoglycaemia, including driving, physical and recreational activity, travel and work productivity.^2^ Furthermore, it can diminish quality of sleep and cause chronic fatigue, with an overall reduction in health‐related quality of life.^1^, ^2^, ^3^ The resulting fear of hypoglycaemia may cause some individuals to deliberately maintain undesirable hyperglycaemia to minimize the risk and severity of further hypoglycaemia events.^2^ Hyperglycaemia is also an important consideration in the management of individuals with diabetes, with poor glycaemic control being associated with increased risk of micro‐ and macrovascular complications, cardiovascular (CV) risk and all‐cause mortality,^4^, ^5^ as well as being a burden on healthcare resources.^6^

The effective use of insulin requires a sensitive balance between achieving and maintaining glycaemic targets while limiting the risk of hypoglycaemia.^1^ Consequently, the assessment and regulatory approval of insulins have depended largely on evidence of glycaemic efficacy combined with incremental reductions in therapy‐induced hypoglycaemia, utilizing data commonly derived from randomized controlled trials (RCTs).^7^

It is also important to determine whether the hypoglycaemia reductions observed in the tightly regulated conditions observed in RCTs are also observed in real‐life clinical practice, in which patient populations are more diverse and clinical monitoring and support is less extensive compared with an RCT.^8^ The more diverse patient populations, less rigorous protocols and less intensive patient follow‐up of real‐world evidence (RWE) studies^9^ may be more representative of clinical practice.^10^, ^11^ As such, although the reporting of hypoglycaemia events may be less accurate in RWE studies, observational studies of electronic health records, medical claims and billing data and registries, or prospective RWE studies such as cross‐sectional surveys are needed to provide a complementary source of information concerning the frequency of hypoglycaemia associated with insulins.

The disparities in definitions, methods of assessment and reporting of hypoglycaemia across RCTs and RWE studies, combined with the differences in trial designs, analyses and populations, present significant challenges when comparing different insulin molecules and formulations. These issues can obfuscate the true differences in the safety of glucose‐lowering therapies and may explain the observed inconsistencies across various regulatory and advisory guidelines.

Differences in reporting of BI therapies in the context of hypoglycaemia have been an ongoing challenge, with great variability among early trials of BIs; however, considerable diversity in reporting still exists in the most recent trials of second‐generation BI analogues, which precludes true comparisons of their safety across trials. Although second‐generation BI analogues have demonstrated lower rates of hypoglycaemia as compared with first‐generation BIs,^12^, ^13^, ^14^ the risk of hypoglycaemia has not yet been eradicated and it is therefore important to facilitate interpretation of efficacy and safety among BIs. In addition, as further advances in BI therapies occur, standardization across trials of these newer therapies and technologies would be beneficial, to facilitate interpretation of their hypoglycaemia risk profiles.

Improved understanding of the differences in reporting of hypoglycaemia is required and, ultimately, greater standardization concerning the way hypoglycaemia is defined, measured and analysed would greatly aid the interpretation of the safety of BI therapies across trials. The present review describes the differences in the way hypoglycaemia has been defined, measured and reported in both RCTs and RWE studies, with a focus on the most recent studies of second‐generation BIs. Potential explanations for the diversity observed across studies are discussed. Nocturnal and daytime hypoglycaemia, both non‐severe and severe, in individuals with type 1 and type 2 diabetes (T1DM and T2DM) are explored.

## 2 DIVERSITY OF HYPOGLYCAEMIA ASSESSMENT AND REPORTING IN RANDOMIZED CONTROLLED TRIALS

Until the advent of treat‐to‐target trial designs, insulin titration in clinical trials was undertaken largely at the discretion of the investigator.^15^ The first treat‐to‐target trial was conducted in 2003; this trial design used a pre‐specified algorithm to titrate either insulin glargine 100 U/mL (Gla‐100) or isophane insulin (neutral protamine Hagedorn [NPH] insulin) to achieve and maintain a target fasting plasma glucose of 5.5 mmol/L (100 mg/dL).^16^ In this way, treat‐to‐target trials highlighted the differences in factors such as hypoglycaemia, as blood glucose levels are driven closer to euglycaemia.^17^

To demonstrate the diversity in reporting of hypoglycaemia across RCTs, the present review focuses on two treat‐to‐target trial programmes of second‐generation BI analogues, namely, the BEGIN trials, which compared insulin degludec (IDeg) with insulin glargine 100 U/mL (Gla‐100),^18^, ^19^, ^20^, ^21^, ^22^, ^23^, ^24^, ^25^, ^26^ and the EDITION trials, in which insulin glargine 300 U/mL (Gla‐300) and Gla‐100 were compared. Some of the older treat‐to‐target trials of first‐generation BI analogues,^12^, ^27^, ^28^, ^29^, ^30^, ^31^ Gla‐100 and insulin detemir (IDet) vs NPH insulin have been included for comparison.^16^, ^32^, ^33^, ^34^

The BEGIN and EDITION trials (Table 1 and Table S1) shared certain common design features. For example, both trials were randomized, open‐label and treat‐to‐target trials. However, there were key differences between the trials, such as the starting dose of insulin, titration algorithms, targets for self‐monitored blood glucose (SMBG) and hypoglycaemia definitions. These differences are examined in greater detail in the following sections.

**Table 1:** Examples of key treat‐to‐target diabetes trials showing disparities in protocol design and hypoglycaemia assessment

References	Insulin(s)	Trial length	Titration algorithm	Definitions of non‐severe hypoglycaemia
BEGIN trials^18^, ^19^, ^20^, ^21^, ^22^, ^23^, ^24^, ^25^, ^26^	IDeg vs Gla‐100	26 or 52 wk	**Starting dose:** T2DM: 10 U T1DM: Same as previous dose (unless ≥1 daily injections, whereby dose for Gla‐100 group was reduced by 20%‐30%) **Titration:** Titrated weekly using pre‐breakfast SMBG from previous 3 days T2DM: Target of pre‐meal SMBG of: 3.9 to <5.0 mmol/L (>70‐<90 mg/dL)^18^, ^21^, ^22^ 3.9–4.9 (70‐88 mg/dL) (BEGIN once long)^19^ <5.0 (<90 mg/dL) (BEGIN low volume)^20^ T1DM: Bolus doses were titrated to pre‐breakfast SMBG of 3.9 to <5.0 mmol/L (70‐<90 mg/dL)^24^	Confirmed hypoglycaemia episodes included those with BG <3.1 mmol/L (<56 mg/dL) or severe (requiring assistance) Severe events were assessed separately Nocturnal hypoglycaemia: 00:01 to 05:59 h
EDITION trials^12^, ^27^, ^28^, ^29^, ^30^, ^31^	Gla‐300 vs Gla‐100	26 wk with a 26‐wk safety extension	**Starting dose**: T1DM and T2DM: Same as previous dose unless NPH was used, whereby a 20% reduction in total daily dose was implemented **Titration:** T2DM: Target fasting SMBG of 4.4 to 5.6 mmol/L (80‐100 mg/dL). Dose was adjusted weekly, based on median fasting SMBG from the preceding 3 days T1DM: Target pre‐breakfast SMBG of 4.4–7.2 mmol/L (80–130 mg/dL)	ADA‐definitions. Confirmed or severe (including any confirmed symptomatic or asymptomatic and/or severe event); documented symptomatic and severe. Threshold was ≤3.9 mmol/L (≤70 mg/dL) and pre‐planned analysis of <3.0 mmol/L (<54 mg/dL) Nocturnal hypoglycaemia: 00:00‐05:59 h)
The treat‐to‐target trial^16^	Gla‐100 vs NPH insulin	24 wk	T2DM patients only **Starting dose:** 10 U **Titration:** Target fasting SMBG of ≤5.6 mmol/L (≤100 mg/dL). Doses were adjusted weekly based on mean SMBG from the previous 2 days	≤4.0 mmol/L (≤72 mg/dL) or severe hypoglycaemia (requiring assistance) and BG <3.1 mmol/L (<56 mg/dL) or recovery following carbohydrate administration. Nocturnal hypoglycaemia defined as events occurring after bedtime insulin dose and before breakfast/morning SMBG or administration of glucose‐lowering agent
IDet vs NPH insulin trials^32^, ^33^, ^34^	IDet vs NPH insulin	20 wk, 26 wk or 2 years	**Starting dose:** T2DM: 10 U^32^ IDet/NPH insulin 40% and insulin aspart 60%, of the total insulin dose before randomization^34^ T1DM: Same as previous dose (unless twice‐daily injections, whereby dose was reduced by 30%^33^) **Titration:** T2DM: Prebreakfast or predinner (depending on whether injections occurred in the morning or the evening) SMBG <6.0 mmol/L (<108 mg/dL). Based on the average of three SMBG readings^32^ IDet/NPH insulin prebreakfast plasma glucose ≤6.1 mmol/L (≤110 mg/dL); aspart 90‐min postprandial glucose level of ≤10.0 mmol/L (≤180 mg/dL). Based on the average of three different SMBG readings^34^ T1DM: BI prebreakfast or predinner SMBG <6.0 mmol/L (<108 mg/dL) Insulin aspart was titrated to a post‐prandial SMBG of ≤9.0 mmol/L (≤162 mg/dL)^33^	Major (requiring third‐party assistance, no SMBG measure required) or confirmed [plasma glucose <3.1 mmol/L (<56 mg/dL)]. Nocturnal hypoglycaemia: 23:00–06:00^32^ Symptomatic hypoglycaemia with a blood glucose value <4.0 mmol/L (<72 mg/dL) or any single plasma glucose value <3.1 mmol/L (<56 mg/dL)^34^ Nocturnal hypoglycaemia defined as events occurring between bedtime and pre‐breakfast SMBG measurement Major (assistance required), minor (plasma glucose <3.1 mmol/L [<56 mg/dL] and individual treated the episode him/herself), or symptoms only (if episodes were not confirmed by a glucose measurement and no assistance was required). Nocturnal hypoglycaemia: 11:00 pm to 6:00 am 33

Abbreviations: ADA, American Diabetes Association; BG, blood glucose; Gla‐100, insulin glargine 100 U/mL; Gla‐300, insulin glargine 300 U/mL; IDeg, insulin degludec; IDet, insulin detemir; NPH, neutral protamine Hagedorn; SMBG, self‐monitored blood glucose; T1DM, type 1 diabetes; T2DM, type 2 diabetes; wk, weeks.

### 2.1 Eligibility criteria

Variability in inclusion and exclusion criteria can influence hypoglycaemia risk results, as various baseline characteristics can be associated with hypoglycaemia.^35^ The BEGIN and EDITION trials attempted to account for differing hypoglycaemia risk factors by grouping trial populations according to the nature of previous antihyperglycaemic therapy (eg, BEGIN basal‐bolus type 2,^18^ BEGIN Low Volume^20^ and the EDITION 1, 2 and 3 trials),^27^, ^28^, ^29^ or by racial background (eg, the BEGIN Asia trial^21^ and the EDITION JP1 and JP2 trials).^30^, ^31^ However, while this demonstrates the efficacy of BIs within specific populations, it complicates interpretation across trials. Large patient‐level meta‐analyses have been performed on the EDITION^14^ and BEGIN^13^ populations, which contribute to comparison of BIs among participants with a range of baseline characteristics, but the post hoc nature of these studies is a significant limitation.

### 2.2 Hypoglycaemia classification and glycaemic thresholds

Various advisory groups have proposed definitions of hypoglycaemia to standardize the way it is reported. However, important heterogeneity exists among these guidelines (Table 2), which has contributed to differences in the way hypoglycaemia has been reported across clinical trials.^7^ A meta‐analysis of trials included in the Canadian Agency for Drugs and Technologies in Health Reports examined the way hypoglycaemia was reported in trials of glucose‐lowering drugs, including oral antihyperglycaemic drugs, fast‐acting insulins and BIs.^36^ This revealed that definitions of hypoglycaemia were included in only 60% of these trials,^36^ and few of these definitions followed American Diabetes Association (ADA)^37^ and European Medicines Agency (EMA)^38^ recommendations for definition of hypoglycaemia as blood glucose (BG) of ≤3.9 mmol/L (≤70 mg/dL), or <3.1 mmol/L (<56 mg/dL), which was recommended by the EMA prior to 2012.^36^

**Table 2:** Summary of changes in regulatory guidelines for reporting hypoglycaemia (adapted from Klonoff et al. 2017^7^)

Advisory group	Year	Recommendations
EMA guidelines on clinical investigation of medicinal products in the treatment of diabetes mellitus^41^	2002	Hypoglycaemia threshold set at <3.0 mmol/L (<54 mg/dL) Categories of hypoglycaemia: **Major**: Requiring external assistance because of severe impairment in consciousness or behaviour with BG <3.0 mmol/L (<54 mg/dL) **Minor:** Symptomatic episode with BG <3.0 mmol/L (<54 mg/dL) without need for assistance, or asymptomatic episode with BG <3.0 mmol/L (<54 mg/dL) **Episodes suggestive of hypoglycaemia**: Hypoglycaemia symptoms without corresponding BG measurement.
ADA – Workgroup on Hypoglycaemia^44^	2005	Hypoglycaemia threshold set at BG ≤3.9 mmol/L (≤70 mg/dL) Categories of hypoglycaemia: **Severe**: Any event requiring aid of another person (recorded blood glucose not required) **Documented symptomatic**: Symptomatic event followed by BG of ≤3.9 mmol/L (≤70 mg/dL) **Asymptomatic**: BG of ≤3.9 mmol/L (≤70 mg/dL) without symptoms **Probable symptomatic**: Symptomatic event without BG reading **Relative**: Hypoglycaemia symptoms but BG of >3.9 mmol/L (>70 mg/dL) At a minimum, incidence and event rates should be reported for first three classifications
FDA draft guidance for development of diabetes therapeutics^97^	2008	Recommended use of ADA guidelines
EMA guidelines on clinical investigation of medicinal products in the treatment of diabetes mellitus^98^	2010	Reaffirmed guidelines set by EMA in 2002 with further definition of severe hypoglycaemia: Severe hypoglycaemia involves central nervous system dysfunction without any other apparent cause, which is reversible by administration glucagon or glucose; requires aid of another
EMA guidelines on clinical investigation of medicinal products in the treatment of diabetes mellitus^38^	2012	Updated recommendations to align with ADA guidelines. Abandoned “Major” and “Minor” terms Categories of hypoglycaemia: **Severe:** Any event requiring aid of another person (recorded blood glucose not required) **Documented symptomatic:** Symptomatic event followed by BG of ≤3.9 mmol/L (≤70 mg/dL) **Asymptomatic:** BG of ≤3.9 mmol/L (≤70 mg/dL)
ADA Workgroup of the American Diabetes Association and the Endocrine Society^99^	2013	Reaffirmed guidelines set by ADA in 2005; however, “relative hypoglycaemia” was re‐termed “pseudo‐hypoglycaemia”
The International Hypoglycaemia Study Group^39^, ^40^	2017	Consensus of this group stated that a hypoglycaemia threshold indicative of clinically significant hypoglycaemia was required, which needed to be avoided because of its immediate and long‐term danger to individuals. This group presented the below threshold recommendations: **Level 1** (≤3.9 mmol/L [≤74 mg/dL]): Glucose alert value; sufficiently low to warrant intervention **Level 2** (<3.0 mmol/L [<54 mg/dL]): Clinically significant hypoglycaemia **Level 3** (no specific glucose threshold): Severe hypoglycaemia
ADA standards of care in diabetes^37^	2017	ADA standards of care guidelines were updated in 2017 to reflect the position statement released by the International Hypoglycaemia Study Group

Abbreviations: ADA, American Diabetes Association; BG, blood glucose; BI, basal insulin; EMA, European Medicines Agency; FDA, Food and Drug Administration.

The differences in BG thresholds for non‐severe hypoglycaemia can be seen in the examples of treat‐to‐target trials shown in Table 1 and Table S1. Indeed, trials within both the BEGIN and EDITION programmes consistently used the same BG thresholds for non‐severe hypoglycaemia, but these thresholds differed between programmes.

Interpretation of hypoglycaemia frequency is difficult when trials have employed very study‐specific, non‐standard or composite endpoints for assessing hypoglycaemia. For example, both the EDITION^12^, ^27^, ^28^, ^29^, ^30^, ^31^ and BEGIN^18^, ^19^, ^20^, ^21^, ^22^, ^23^, ^24^, ^25^, ^26^ trials utilized a composite endpoint of confirmed or severe hypoglycaemia.

The disparate nature of the definitions of hypoglycaemia applied in RCTs of BIs highlights the need for greater consensus and standardization in the measurement of hypoglycaemia outcomes. Using more stringent BG cut‐offs (eg, <3.0 mmol/L [<54 mg/dL] or <3.1 mmol/L [<56 mg/dL]) will generate lower frequencies of clinically relevant non‐severe hypoglycaemia, but the glycaemic threshold no greater than 3.9 mmol/L (≤70 mg/dL), now designated as an “alert level,” has been considered for some time to be indicative of non‐severe hypoglycaemia.^39^, ^40^ Ideally, in line with recent guidelines, both thresholds should be reported routinely.^39^, ^40^

Consistency in the definitions of non‐severe and severe hypoglycaemia episodes in clinical trials is not just desirable, it is essential in facilitating interpretations of the frequency of hypoglycaemia among different treatment regimens. This should be possible with adoption of the revised definitions of hypoglycaemia that have achieved international consensus.^39^, ^40^ For instance, definitions of severe hypoglycaemia varied considerably before the initial ADA working group guidelines were published in 2002,^41^, ^42^, ^43^ including definitions in the original treat‐to‐target trial.^16^ However, severe hypoglycaemia has typically been defined across all trials as an event requiring external assistance to administer oral carbohydrate or parenteral therapy in the form of intramuscular glucagon or intravenous dextrose, regardless of whether BG has been measured, as per ADA recommendations.^44^

### 2.3 Duration of follow‐up

The duration of patient follow‐up in RCTs of BIs has varied widely, ranging from 4 weeks^45^, ^46^ to 2 years,^33^ and is apparent in the treat‐to‐target trials shown in Table 1 and Table S1. Variation in trial length is an important factor in the quantification of event frequency. Longer trials increase the probability of observing hypoglycaemia events, which is particularly important for those classified as severe, thereby mitigating the potential for false negative results and enhancing the statistical power to detect differences among BIs in therapy‐induced hypoglycaemia, assuming that the effect sizes remain constant. If participants withdraw from a study because of morbidity associated with severe hypoglycaemia, the risk of exposure to hypoglycaemia is modified by this attrition, particularly if these are individuals with the highest risk of hypoglycaemia. As such, the proportion of participants experiencing at least one hypoglycaemia event may be more relevant in shorter trials, as this measure would be greatly influenced by the progressive withdrawal of individual participants during a prolonged follow‐up period. Additionally, in trials that use retrospective patient recall of hypoglycaemia, the period over which hypoglycaemia events are recalled can impact upon the accuracy of hypoglycaemia frequency. The recall of non‐severe hypoglycaemic events is inaccurate with a recall period of more than 1 week; however, the accuracy of severe hypoglycaemia recall does not appear to be affected by longer recall periods.^47^

Alternatively, event rates of hypoglycaemia could be used; however, in trials with a duration of less than 1 year, annualized event rates are estimated from extrapolation of recorded data. Examples of this include the initial treat‐to‐target trial,^16^ the EDITION trials^12^, ^27^, ^28^, ^29^, ^30^, ^31^ and the BEGIN Low Volume,^20^ BEGIN FLEX,^22^ BEGIN Asia^21^ and BEGIN basal‐bolus T1DM^23^ trials. Often, these trials include safety extension follow‐ups, whereby hypoglycaemia events continue to be recorded, such as the 6‐month safety extensions of the EDITION trials.^48^, ^49^, ^50^, ^51^, ^52^ However, participants often maintain BI doses during these extension periods, and, in efforts to avoid hypoglycaemia, they may be less effective in titrating their dose and achieving glycaemic targets.

### 2.4 Clock time definitions

Nocturnal hypoglycaemia has negative effects on quality of life^3^ and can incur major economic costs.^53^ In clinical trials, nocturnal hypoglycaemia is often defined by its occurrence during a pre‐determined clock time, which differs among studies. The longer the period selected to define night‐time, during which it is assumed that participants are asleep, the higher the potential rate of nocturnal hypoglycaemia. Most treat‐to‐target trials of BIs define this nocturnal period as approximately 12:00 am to 6:00 am, as this interval avoids confounding factors not related to BI therapy. However, variability in the nocturnal interval used still exists across trials, with older trials making a single SMBG measurement at 3:00 am,^45^, ^46^ while other trials have used 10:00 pm to 7:00 am 54 or 11:00 pm to 6:00 am.^33^, ^55^ While trials using earlier cut‐off times for the end of the nocturnal interval may potentially underestimate the true impact of nocturnal hypoglycaemia, this decreases the chance of capturing hypoglycaemia induced by prandial insulin use during breakfast in individuals receiving basal‐bolus regimens.

While the standard definition of 12:00 to 6:00 am appears to have been adopted by most trials, this definition still has limitations; for instance, it may lead to mis‐categorization of hypoglycaemia events as nocturnal in individuals with different patterns as a result of shift‐work. Additionally, meta‐analyses of the BEGIN and EDITION trials explored the effect of using different time intervals to estimate nocturnal hypoglycaemia (BEGIN original definition, 12:01 am to 5:59 am vs expanded definitions, 9:59 pm to 5:59 am or 12:01 am to 7:59 am; EDITION original definition, 12:00 am to 05:59 am vs the expanded definition, 22:00 to pre‐breakfast SMPG measurement).^56^, ^57^ Both of these analyses highlighted that a large number of hypoglycaemia events occurred within the pre‐breakfast time period.^56^, ^57^ As such, the commonly used nocturnal window of 12:00 am to 6:00 am may lead to underestimation of the clinically relevant impact of nocturnal hypoglycaemia. Furthermore, in meta‐analysis of the BEGIN trials, the hypoglycaemia risk benefit of IDeg vs Gla‐100 was significant for all comparisons, with the exception the 00:01 to 07:59 h nocturnal interval.^56^, ^57^

### 2.5 Titration protocols

As discussed earlier, the advent of the treat‐to‐target trial design facilitated comparison of safety profiles, including hypoglycaemia, among BIs. Nevertheless, differences in various clinical trials using the treat‐to‐target design are apparent (Table 1 and Table S1). One such example is a design in which different target BG concentrations have been used. While no correlation between target BG and HbA1c has been shown,^15^ adopting a lower target BG may be more likely to increase both the incidence and prevalence of hypoglycaemia events, which may enhance the prospect of observing statistically significant differences among treatments.

**Figure 1:**
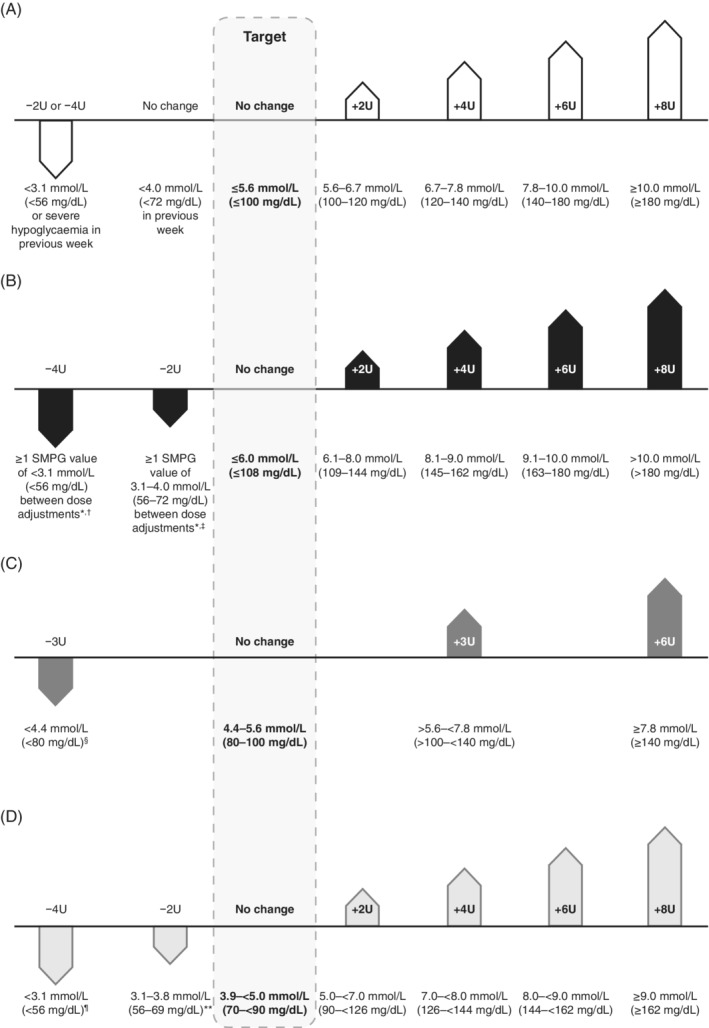
Examples of different titration algorithms used in T2DM for A, Gla‐100 vs NPH; B, IDet vs NPH; C, Gla‐300 vs Gla‐100 (EDITION); D, IDeg vs Gla‐100 (BEGIN). Algorithms shown were taken from the original treat‐to‐target trial (Gla‐100 vs NPH),^16^ IDet vs NPH,^32^ the EDITION 1, 2 and 3 trials (Gla‐300 vs Gla‐100),^27^, ^28^, ^29^ and the BEGIN Once Asia and BEGIN Flex trials (IDeg vs Gla‐100).^21^, ^22^ Dose adjustments were made based on the median fasting SMBG value of the previous three consecutive days (or previous two consecutive days for the original treat‐to‐target trial), unless otherwise stated. ^a^Dose decreased unless there was an obvious reason for the low BG value; ^b^for doses >40 U, dose was reduced by 10%; ^c^for doses >40 U, dose was reduced by 5%; ^d^if SMBG was <3.3 mmol/L (<60 mg/dL), insulin dose could be reduced by ≥3 units, at the investigators discretion; ^e^for doses >45 U, a 10% reduction was recommended; ^f^for doses >45 U, a 5% reduction was recommended

The frequency of insulin dose adjustments and the incremental changes during titration have also differed among trials. Although the BEGIN and EDITION trials typically used the same titration algorithm for control and experimental insulins within each trial, the BEGIN trials utilized a titration algorithm with larger dose adjustments than that utilized in the EDITION trials (Figure 1), which may have affected the incidence and prevalence of hypoglycaemia events during the titration period of the former.^12^, ^13^, ^14^, ^18^, ^19^, ^20^, ^21^, ^22^, ^23^, ^24^, ^25^, ^26^, ^27^, ^28^, ^29^, ^30^, ^31^

### 2.6 Study periods

The timing and duration of a clinical trial influences the frequency of hypoglycaemia, and its clinical relevance should be interpreted in relation to the period of assessment. During the first few months of a new therapy, some recipients will experience problems. Indeed, approximately 5% of individuals with T2DM experience hypoglycaemia within 6 months of initiation of insulin therapy.^58^ When hypoglycaemia occurs soon after insulin therapy is commenced, individuals are more likely to discontinue BI therapy within the first 12 months (hazard ratio, 1.16; 95% CI, 1.03, 1.32; *P* = .016).^58^ Although no studies are available for direct illustration, reducing hypoglycaemia risk during the titration period, when most dose adjustments are made, may potentially improve and facilitate adherence to, and persistence with, the regimen. In the EDITION trials, the hypoglycaemia benefit with Gla‐300 over Gla‐100 was often more pronounced during the titration period, as analysed during the first 8 weeks.^59^

Time beyond the titration period, often referred to as the maintenance period, also has ongoing clinical importance. During the maintenance period, it is important that insulin dose adjustments are continued, albeit less intensively than during the titration period, to ensure that patients maintain glycaemic targets and avoid hypoglycaemia; as such, this period may represent the ongoing clinical reality for patients using BIs. It is, therefore, important to ascertain whether there are extended benefits in reduction of hypoglycaemia during the maintenance period, as this may have implications for long‐term patient adherence. A meta‐analysis of the BEGIN trials demonstrated that, while the risk of hypoglycaemia during the titration period was similar for IDeg and Gla‐100, a risk reduction was observed with IDeg vs Gla‐100 during the maintenance period in individuals with T2DM (relative risk, 0.75; 95% CI, 0.66‐0.87). This benefit translated to lower hypoglycaemia risk throughout the period of treatment.^13^

Extension periods examine efficacy and safety over a prolonged period of exposure to therapies and are often more representative of an RWE clinical setting; the follow‐up is less rigorous, and participants are less likely to conform to the closely controlled treatment protocols of clinical trials. Consequently, the apparent hypoglycaemia risk benefits that are observed during the on‐study period in RCTs may be less pronounced in the extension period. For instance, 12‐month data from the EDITION trials showed that HbA1c levels in the Gla‐100 group increased slightly in the 6‐month extension period, which coincided with slight attenuation of the hypoglycaemia benefit of Gla‐300 over Gla‐100.^59^ A possible explanation may be that insulin dose was titrated less rigorously during the extension period, thereby reducing the overall risk of hypoglycaemia, but at the cost of achieving less stringent glycaemic control. In the BEGIN Once Long trial, HbA1c levels also rose in the extension period, with similar increments being observed for IDeg and Gla‐100; however, the hypoglycaemia risk benefit seen with IDeg vs Gla‐100 improved during this period.^60^

### 2.7 Hypoglycaemia measurements

Hypoglycaemia events in RCTs are typically confirmed by a BG measurement, usually using SMBG. However, because SMBG testing causes discomfort to the participants, measurements are made relatively infrequently. As such, SMBG cannot provide an accurate record of the glucose fluctuations that occur in an individual on an ongoing basis. Furthermore, SMBG is not a practical method of detecting hypoglycaemia that occurs during sleep and would require that individuals were awakened, either by the hypoglycaemia event itself, which typically result in the SMBG‐reported nocturnal events in RCTs, or at intervals during the night in order for hypoglycaemia events to be recorded.

Continuous glucose monitoring (CGM) measures interstitial tissue glucose, providing real‐time glucose measurements throughout the day and night.^61^ This enables a large amount of data to be collected, and may facilitate a more detailed analysis of the frequency of biochemical, and asymptomatic, hypoglycaemia.^62^ As CGM provides information concerning glucose fluctuations over time, it is a valuable tool for comparing the pharmacodynamic profiles of second‐generation BI analogues, and allows identification of the time they are most likely to induce hypoglycaemia. However, it is not possible to compare trials using different methods of monitoring glucose and there is a paucity of published studies that have used CGM to investigate the efficacy and safety of insulins. Studies that have utilized CGM have generally been part of a sub‐study of relatively few patients and, because CGM is data rich, statistical differences in hypoglycaemia frequency may be apparent, which are not evident with SMBG.

CGM has several limitations. Initially, there were concerns regarding its accuracy, with early devices having a variability of more than 20%; variability has since improved to approximately 10%.^62^ Furthermore, aside from sensor issues, for example, occasional sensor failure and the requirement for regular recalibration with SMBG, consensus is currently lacking as to which of the glycaemic parameters derived from CGM should be reported.^63^, ^64^, ^65^ The absence of system comparability, combined with data communication systems that are constantly evolving, also prevent comparisons between different CGM trials.^65^

CGM has been used in RCTs to compare Gla‐100 and IDet,^66^ and IDeg, in combination with insulin aspart,^67^ and Gla‐300;^68^ however, such trials predominantly focus on comparisons of glycaemic variability. For example, the study by Bergenstal et al., which used CGM to compare Gla‐100 and Gla‐300, defined hypoglycaemia events using only the traditional methods of SMBG confirmation.^68^ Furthermore, the diversity in glycaemic parameters used to measure low blood glucose levels is apparent across these trials, with Bergenstal et al. measuring only the time below certain glycaemic thresholds,^68^ whereas Liebl et al. measured both the time below thresholds and individual excursions below these thresholds.^67^ Nevertheless, CGM may provide a more comprehensive assessment of hypoglycaemia events in RCTs than SMBG measurements. The opportunity to include routine CGM is increasing steadily, particularly as the technology is rapidly advancing.

### 2.8 Descriptive and analytical approaches to frequency estimation

When comparing insulins, the frequency of hypoglycaemia can be reported either by recording the number of participants experiencing at least one event, binary outcome or incidence, or by recording the number of total events per participant‐year, count variable or annualized event rates. Presenting both outcomes is important, to provide an accurate representation of hypoglycaemia events. If, for example, one individual experienced multiple hypoglycaemia events, this may under‐ or over‐represent the binary and count variable outcomes. In line with ADA recommendations,^37^, ^44^ many RCTs report both the binary outcome and the count variable analyses of hypoglycaemia (Table 1 and Table S1). However, sometimes only one analysis of between‐treatment differences has been reported, often the hypoglycaemia event rate ratios,^54^, ^55^, ^69^, ^70^ which hinders assessment of the consistency of results across trials. For instance, the BEGIN trials^18^, ^19^, ^20^, ^21^, ^22^, ^23^, ^24^, ^25^, ^26^ focused on rate ratios between BIs, whereas the EDITION studies^12^, ^27^, ^28^, ^29^, ^30^, ^31^ presented both the rate ratio and relative risks.

The methods used to assess these outcomes and adjustments for confounding factors can also vary across trials. For instance, the BEGIN studies employed a negative binomial regression model to assess the rates of hypoglycaemia, with treatment, antihyperglycaemic therapy at screening, gender and geographical region as fixed factors, and with age as a covariate,^18^, ^19^, ^20^, ^21^, ^22^, ^23^, ^24^, ^25^, ^26^ whereas the EDITION trials utilized an overdispersed Poisson regression model.^12^, ^14^, ^27^, ^28^, ^29^, ^30^, ^31^ However, the EDITION studies also analysed the number of individuals experiencing more than one hypoglycaemia event, a binary outcome, for which a Cochran‐Mantel‐Haenszel method, a statistical method that tests the association of a treatment with a specific binary outcome, was used to assess between‐treatment differences, stratified by HbA1c at screening and by geographical region.^12^, ^27^, ^28^, ^29^, ^30^, ^31^ The variation in analyses described above may influence outcomes and, therefore, standardization in statistical testing of hypoglycaemia endpoints should be applied to trials of BIs.

## 3 HYPOGLYCAEMIA ASSESSMENT IN RWE STUDIES

### 3.1 RCTs versus real‐life clinical practice

RCTs are considered to be the “gold standard” and are essential for demonstrating the efficacy and safety of new therapies.^71^ However, inherent limitations in their design may lead to underestimation of rates of hypoglycaemia.^72^ For example, RCTs may exclude participants with very high HbA1c levels or those with renal impairment,^8^ both of whom are associated with increased risk of hypoglycaemia.^35^ Clinical trial participants are often a more engaged and informed subpopulation of individuals with diabetes and, hence, are more likely to adhere to treatment and to accept advice on diabetes self‐care.^8^ In addition, clinical monitoring and support are much more extensive during an RCT compared with that received within routine clinical practice. While this may ensure more efficient capture of hypoglycaemia events, it limits the extrapolation of data on hypoglycaemia occurrence from clinical trials to influence real‐life practice.^8^

Currently, the hypoglycaemia definitions and glycaemic threshold values used in regulatory guidelines reflect consensus guidelines from working groups; however, the evidence from RWE studies that influence these decisions is limited. Consequently, the rates of hypoglycaemia reported in these trials may not be representative of real‐life. For example, a recent systematic review showed that RWE studies frequently report higher rates of hypoglycaemia than those reported in RCTs, particularly when the primary focus of the study was to investigate hypoglycaemia.^72^

High‐risk populations, such as children, individuals over 70 years of age, pregnant women, individuals with renal impairment or with impaired awareness of hypoglycaemia (IAH), and patients with a history of previous severe hypoglycaemia are often excluded from RCTs. However, it is these vulnerable populations that may experience the greatest benefit from new therapies that confer good glycaemic control with a lower hypoglycaemia risk profile. Attempts to address knowledge gaps in previous RCTs concerning these vulnerable or high‐risk populations have been made by specifically recruiting participants from high‐risk populations such as children,^43^, ^54^, ^73^ pregnant women,^74^, ^75^, ^76^ older individuals^77^ and individuals with specific risk factors for hypoglycaemia.^78^, ^79^ However, these trials all involved a high level of participant follow‐up and employed strict protocols for titration; as such, they may not represent real‐life clinical experience.

### 3.2 Real‐world evidence studies

RWE studies include diverse participant populations, use less strict protocols for administering therapies, involve less frequent contact and communication with participants^9^ and, thus, help to bridge the gap between regulatory trials and clinical practice. Specifically, retrospective observational studies of clinical databases and electronic healthcare registries, or databases of health insurance claims in the USA, can be valuable sources of data. Such registries include the TEENs study, which investigated factors affecting glycaemic control in children, teens and young adults with T1DM,^80^ the T1D Exchange clinic registry^81^ and the UK Clinical Practice Research Datalink (CPRD).^82^ The effectiveness and safety of Gla‐100 or IDeg have been investigated in observational retrospective studies of such databases, such as the DELIVER^83^, ^84^, ^85^ and ReFLECT studies.^86^ Additionally, the LIGHTNING and CONFIRM trials compared rates of hypoglycaemia between Gla‐300 and IDeg reported in real‐world data from electronic healthcare records.^87^, ^88^

Given the less stringent protocols that are often associated with RWE studies, the diversity in hypoglycaemia reporting in these studies is likely to be even greater than that in RCTs. Hypoglycaemia documentation in electronic medical record/registry studies is often determined by self‐reporting by individuals to their physicians, because of the minimal participant follow‐up employed in most RWE studies. This could lead to under‐reporting of events in retrospective observational RWE studies, and makes results difficult to interpret. Self‐reported hypoglycaemia may not be accompanied by confirmatory BG measurements or by a clear description of the event. Additional confounding factors include IAH, failure of individuals to disclose hypoglycaemia events to healthcare professionals (HCPs), in some cases because this may have unwanted consequences such as the risk of losing a driving licence, incorrect documentation of hypoglycaemia episodes by HCPs, failure to recall all hypoglycaemia events that patients have experienced, and neglect on the part of HCPs to enquire about previous hypoglycaemia.^2^, ^89^, ^90^ Additionally, clinical registries and other electronic healthcare records use coded medical records, with several coding systems available, and the risk of miscoding, or potential lack of coding, can lead to inaccurate or inappropriate case selection.^90^, ^91^ However, the recent development of Natural Language Processing enables hypoglycaemia events to be identified from clinical notes, with one study demonstrating a large increase in the number of reported events (133% for any form of hypoglycaemia) in individuals with T2DM; the increase was particularly evident in reports of non‐severe hypoglycaemia.^92^

Despite their limitations, retrospective observational RWE studies of diabetes and hypoglycaemia are extremely useful for generating data in settings that more closely resemble clinical practice. Furthermore, given that many observational studies are less resource‐intensive, the capacity for long periods of follow‐up is much greater than that for many RCTs. One such example is the UK Prospective Diabetes Study (UKPDS), which examined the frequency of self‐reported hypoglycaemia of differing severities in individuals with T1DM and T2DM over a follow‐up period of 10 years.^93^ This study highlighted the observation that rates of hypoglycaemia in unselected participant populations were often higher than those reported in RCTs.^93^

RWE studies are also able to demonstrate the epidemiology and economic burden associated with hypoglycaemia. For example, the global Hypoglycaemia Assessment Tool (HAT) study estimated the frequency and severity of hypoglycaemia worldwide and demonstrated that hypoglycaemia rates were very high, with large variations observed between geographical regions.^94^ The HAT study also showed that prospective recording demonstrated much higher rates of hypoglycaemia than retrospective assessment. Meneghini et al.^95^ reported results from a retrospective observational study which highlighted that, compared with absence of hypoglycaemia, severe hypoglycaemia was associated with significantly lower health‐related quality‐of‐life (*P* < .001), reduced work productivity (*P* = .004), impaired ability to perform regular daily activities (*P* < .001), greater healthcare‐resource utilization and increased total healthcare costs (*P* < .001).^95^ The DELIVER‐2 study provided valuable insight into cost‐effectiveness and healthcare resource utilization associated with hypoglycaemia risk in individuals receiving BIs.^83^

Prospective observational studies offer an alternative approach to retrospective observational studies of electronic health records and registries. One example is the DUNE study, which assessed the association between achieving a pre‐determined HbA1c target and the frequency of hypoglycaemia in a real‐life setting in individuals with T2DM.^96^ The prospective design of the study enabled the use of three hypoglycaemia categories for reporting events: severe (any event requiring assistance); non‐severe (any event associated with typical symptoms, regardless of BG measurement); and documented [any event with a BG measurement either ≤3.9 mmol/L (≤70 mg/dL) or <3.0 mmol/L (<54 mg/dL)].^96^ This enabled more rigorous definitions of hypoglycaemia to be applied while still utilizing a real‐life clinical practice setting. Evidence from the DUNE study highlighted the observation that achievement of glycaemic targets with BIs was poor (~30%) in a real‐life setting, possibly because of suboptimal titration of insulin.^96^ Interestingly, the DUNE study also showed that HbA1c targets were more likely to be achieved in patients who experienced more hypoglycaemia events.^96^ Second‐generation BI analogues, such as Gla‐300 and IDeg, enable individuals with diabetes to achieve similar levels of glycaemic control, with a lower risk of hypoglycaemia, as compared to first‐generation BI analogues; however, it is unknown whether individuals will titrate these insulins more rigorously in a real‐life setting. Pragmatic RWE studies, which seek to compare the effectiveness of two or more interventions in a real‐world setting, may also provide a more realistic indication of therapeutic effectiveness as compared to explanatory RCTs.

RWE studies can be valuable for the assessment and comparison of hypoglycaemia in an everyday setting that resembles routine clinical practice. However, as with RCTs, consensus concerning the way hypoglycaemia should be reported in RWE studies is required to facilitate interpretations of hypoglycaemia frequencies to inform clinical decision making.

## 4 CONCLUSIONS

The careful balance of optimal glycaemic control, with avoidance of hypoglycaemia, remains an ongoing issue, even with newer second‐generation BI analogues that are associated with lower hypoglycaemia risk. It would therefore be advantageous to compare the hypoglycaemia risk benefits of different BI therapies across studies; however, the differences in BG threshold, trial design statistical analyses, and definitions used to report hypoglycaemia across RCTs and RWE studies present major issues for such comparisons.

Consensus in international guidelines is required to standardize the way hypoglycaemia is reported across both RCTs and RWE studies; such guidelines would help to avoid diversity in hypoglycaemia reporting and to facilitate interpretations of hypoglycaemia risk between different BI therapies. These guidelines should specify aspects such as definitions of hypoglycaemia, choice of BG thresholds, study length, including titration, maintenance and extension periods in RCTs and the duration of patient follow‐up in RWE studies, titration algorithms, glycaemic targets, and the statistical methods that should be used to analyse hypoglycaemia.

In this review, promising advances towards standardization are highlighted, some of which have already been achieved. For example, an important step in standardizing the definitions of hypoglycaemia has been taken with the recent recommendations made by the International Hypoglycaemia Study Group, which have been accepted by the ADA and EASD.^39^, ^40^ Additionally, studies increasingly use a standard window of 12:00 to 6:00 am to define nocturnal hypoglycaemia.

Consensus guidelines concerning the way to measure and report hypoglycaemia events detected by CGM would be beneficial and can be feasibly achieved, but they will require large trials investigating the use of CGM as SMBG to detect hypoglycaemia, which would enable evidence‐based opinion concerning the utility of CGM devices and the optimal glycaemic parameters for reporting in CGM‐based studies.

However, standardization may not be easily achieved with certain aspects of study design. Inclusion criteria in RCTs may vary, to investigate efficacy and safety in specific populations such as individuals with T2DM undergoing basal‐bolus regimens. While this helps to eliminate potential confounders, it also makes interpretation among trials difficult. One approach may be to reduce the number of inclusion criteria and to include more varied populations, similar to those experienced in clinical practice; however, this would require more complex statistical methods to account for potential bias.

While the treat‐to‐target trial design has facilitated comparisons between BIs for factors other than glycaemic control, including hypoglycaemia, substantial variation in glycaemic targets among trials remains common. Given that the ADA recommends different targets in vulnerable populations, such as children or frail elderly individuals,^37^ several standardized glycaemic targets may be required for different age groups or for patients at high risk of hypoglycaemia. The variation observed in titration algorithms among different treat‐to‐target trials may also be a consequence of the different pharmacokinetics of various BIs.

Study length of RCTs comparing BIs may vary, based on the purpose of the trial, with phase 1 and 2 trials being shorter proof of concept trials, and the length can also be determined by the durability of endpoints of interest. However, it is feasible that study length could be standardized according to the clinical trial phase. Additionally, standardization of the length of “titration” phases would facilitate interpretations of hypoglycaemia frequency during the period in which individuals are initiating new BIs and hypoglycaemia is more frequent.

Despite the clinical importance of hypoglycaemia, studies of BIs have focused mainly on glycaemic control as primary endpoint, with hypoglycaemia remaining a secondary endpoint. While this complies with the requirements of regulatory agencies, it may contribute to the observed diversity in the way hypoglycaemia is reported across studies of BIs. Future studies that investigate hypoglycaemia as a primary endpoint would be of considerable value and interest, particularly if such studies were designed according to future consensus guidelines on hypoglycaemia reporting. This may also facilitate standardization, to some degree, of the statistical approaches to assessment of hypoglycaemia. While standardization may not yet be feasible across all trials assessing BIs, greater consistency among trials would allow clinically important meta‐analyses to be undertaken to compare various basal insulins.

The present review has some limitations. It was not a systematic meta‐analysis and, as such, some recent trials of second‐generation Bis may have been omitted. In addition, this review focuses on a small subset of diabetes therapies, specifically second‐generation BI analogues. However, the noted disparities in hypoglycaemia reporting are also apparent in trials of various antihyperglycaemic drugs.

In conclusion, hypoglycaemia risk profiles of BIs remain important factors in choosing between therapies, but the current diversity in the way hypoglycaemia is reported across RCTs and RWE studies prevents comparisons among studies. The development and application of consensus guidelines denoting the way hypoglycaemia should be defined and reported would contribute to future study design in a way that facilitates interpretation of hypoglycaemia risk profiles among BIs across studies.

## CONFLICT OF INTEREST

B.F. has served on Speakers' Bureaus for Eli Lilly, Novo Nordisk, Sanofi, Roche and Merck; and has served on Advisory Boards of Eli Lilly, Novo Nordisk, Locemia Solutions and Zucara. S.H. has served as a consultant to Abbott, AstraZeneca, Boehringer Ingelheim, Eli Lilly, Janssen, Merck, Novo Nordisk and Sanofi; and has received research support from Abbott, AstraZeneca, Boehringer Ingelheim, Eli Lilly, Janssen, Novo Nordisk and Sanofi. A.R.‐L. declares no conflicts of interest.

## AUTHOR CONTRIBUTIONS

The authors were involved in the conception of the review article, the generation of the review outline and all subsequent drafts. All authors critically reviewed the manuscript and approved the final version for submission.

## Supporting information


**File S1.** Supporting Information.
