# Supplementary material for: Reporting of hypoglycaemia in clinical trials of basal insulins: A need for consensus
Source: Diabetes Obes Metab. 2019 Apr 29;21(7):1529–42. doi: 10.1111/dom.13732 (PMC6767397; doi:10.1111/dom.13732)
Supplement: Supplementary file 1 — File S1. Supporting Information. [file DOM-21-1529-s001.docx]

**Supplementary Table 1.** Comparisons of the protocol design and hypoglycaemia assessments across the BEGIN and EDITION treat-to-target diabetes randomised controlled trials

| **Reference** | **Insulin(s)/diabetes type/prior treatment** | **Trial length, weeks** | **Titration algorithm** | **Definitions of non-severe hypoglycaemia** |
| --- | --- | --- | --- | --- |
| **BEGIN trials** | | | | |
| BEGIN BB type 2 NCT00972283  Garber *et al*. Lancet 2012; 379: 1498–507^1^ | IDeg-100 vs  Gla-100  T2DM requiring insulin ± OAD | 52 | **Starting dose**  BI naïve: 10 U  Previous OD BI: equivalent dose of Gla-100 or IDeg  Previous BID BI: 20–30% dose reduction of  Gla-100; reduced at investigators discretion for IDeg-100  **Titration**  Titrated weekly based on mean pre-breakfast SMBG from previous three days to target of  3.9–<5.0 mmol/L (70–<90 mg/dL) | **Conﬁrmed**  Plasma glucose <3.1 mmol/L  (<56 mg/dL) (irrespective of symptoms) or severe (requiring assistance)  **Nocturnal**  Between 0001 h and 0559 h (inclusive) |
| BEGIN Once Long NCT00982644 Zinman *et al*. Diabetes Care 2012; 35: 2464–71^2^ | IDeg-100 vs  Gla-100  Insulin-naïve T2DM requiring OAD | 52 | **Starting dose**  10 U  **Titration**  Titrated weekly based on mean pre-breakfast SMBG from previous three days to target of  3.9–4.9 mmol/L (70–88 mg/dL) | **Conﬁrmed**  Plasma glucose <3·1 mmol/L  (<56 mg/dL) (irrespective of symptoms) or severe (requiring assistance, no SMBG confirmation)  **Nocturnal**  Between 0001 to 0559 h (inclusive) |
| BEGIN Low Volume NCT01068665 Gough *et al*. Diabetes Care 2013; 36: 2536–42^3^ | IDeg-200 or  Gla-100  Insulin-naïve T2DM requiring OAD | 26 | **Starting dose**  10 U  **Titration**  Titrated weekly based on mean pre-breakfast SMBG from previous three days to target of  <5.0 mmol/L (<90 mg/dL) | **Conﬁrmed**  Plasma glucose <3.1 mmol/L  (<56 mg/dL) (irrespective of symptoms) or severe (requiring assistance).  **Nocturnal**  Between 0001 to 0559 h (inclusive) |
| BEGIN Once Asia NCT01059799 Onishi *et al*. J Diabetes Invest 2013; 4: 605–12^4^ | IDeg-100 vs  Gla-100  Insulin-naïve T2DM requiring OAD | 26 | **Starting dose**  10 U  **Titration**  Titrated weekly based on mean pre-breakfast SMBG from previous three days to target of  3.9–<5.0 mmol/L (70–<90 mg/dL) | **Conﬁrmed**  Plasma glucose <3.1 mmol/L  (<56 mg/dL) (irrespective of symptoms) or severe (requiring assistance)  **Nocturnal**  Between 0001 to 0559 h (inclusive) |
| BEGIN Flex NCT01006291 Meneghini *et al*. Diabetes Care 2013; 36: 858–64^5^ | IDeg-100 vs  Gla-100  T2DM requiring OADs or prior BI ± OAD | 26 | **Starting dose**  BI naïve: 10 U  Previous OD BI: equivalent dose of Gla-100 or IDeg-100  Previous BID BI: 20–30% dose reduction of  Gla-100; reduced at investigators discretion for IDeg-100  **Titration**  Titrated weekly based on mean pre-breakfast SMBG from previous three days to target of  3.9–<5.0 mmol/L (70–<90 mg/dL) | **Conﬁrmed**  Plasma glucose <3.1 mmol/L  (<56 mg/dL) (irrespective of symptoms) or severe (requiring assistance)  **Nocturnal**  Between 0001 to 0559 h (inclusive) |
| BEGIN BB T1 NCT01074268 Davies *et al*. Diabetes Obes Metab 2014; 16: 922–30^6^ | IDeg-100 vs  IDet (with mealtime insulin aspart)  T1DM requiring BI and prandial insulin | 26 | **Starting BI dose**  Matched to previous OD or total BID BI dose on a 1:1 basis; investigator could adjust start dose to reduce risk of hypoglycaemia.  **Titration**  BI titrated weekly based on mean pre-breakfast SMBG from previous three days to target plasma glucose of 3.9–4.9 mmol/L (70–88 mg/dL)  Insulin aspart was titrated weekly based on three pre-prandial SMBG values. BI titration was prioritised in the first 8 weeks | **Conﬁrmed**  Plasma glucose <3.1 mmol/L  (<56 mg/dL) (irrespective of symptoms) or severe (requiring assistance).  **Nocturnal**  Between 0001 h and 0559 h (inclusive) |
| BEGIN BB T1 long NCT00982228 Heller *et al*. Lancet 2012; 379: 1489–97^7^ | IDeg-100 vs  Gla-100 (with mealtime insulin aspart)  T1DM requiring BI and prandial insulin | 52 | **Starting BI dose**  Previously receiving BI: doses matched to previous BI dose  Previous BID dosing: 20–30% reduction of total daily dose with Gla-100; doses matched to previous dose with IDeg.  **Titration**  BI titrated weekly based on mean pre-breakfast SMBG from previous three days to target of  3.9–<5.0 mmol/L (70–<90 mg/dL)  Insulin aspart titrated to achieve pre-prandial (next meal) and bedtime SMBG target: 3.9–<5.0 mmol/L (70–<90 mg/dL) | **Conﬁrmed**  Plasma glucose <3.1 mmol/L  (<56 mg/dL) (irrespective of symptoms) or severe (requiring assistance).  **Nocturnal**  Between 0001 h and 0559 h (inclusive) |
| BEGIN Flex T1 NCT01079234 Mathieu *et al*. J Clin Endocrinol Metab. 2013; 98(3): 1154–62^8^ | IDeg-100 vs  Gla-100 (with mealtime insulin aspart)  T1DM requiring BI and prandial insulin | 26+26 | **Starting BI dose**  Previous OD BI: equivalent dose of Gla-100 or IDeg-100  Previous BID BI: 20–30% dose reduction of  Gla-100; previous dose matched or reduced at investigators discretion for IDeg-100  **Titration**  BI self-adjusted (Mon, Wed, and Fri) on mean pre-breakfast SMBG from the previous 2–3 days to target of 4.0–5.0 mmol/L (72–90 mg/dL)  Insulin aspart titrated to a mean premeal SMBG target <5.0 mmol/L (<90 mg/dL) | **Conﬁrmed**  Plasma glucose <3.1 mmol/L  (<56 mg/dL) or severe (requiring assistance).  **Nocturnal**  Between 0001 h and 0559 h (inclusive) |
| BEGIN Easy AM NCT01068678 Zinman *et al*. Lancet Diabetes Endocrinol 2013; 1(2): 123–31^9^ | IDeg-200 vs  Gla-100  Insulin-naïve T2DM | 26 | **Starting dose**  10 U (Gla-100 OD)  20 U (IDeg three-times weekly; administered in the morning)  **Titration**  Titrated weekly based on mean pre-breakfast SMBG from previous three days to target of  3.9–<5.0 mmol/L (70–<90 mg/dL) | **Conﬁrmed**  Plasma glucose <3.1 mmol/L  (<56 mg/dL) (irrespective of symptoms) or severe (requiring assistance)  **Nocturnal**  Between 0001 and 0559 h (inclusive) |
| BEGIN Easy PM NCT01076647 Zinman *et al*. Lancet Diabetes Endocrinol 2013; 1(2): 123–31^9^ | IDeg-200 vs  Gla-100  Insulin-naïve T2DM | 26 | **Starting dose**  10 U (Gla-100 OD)  20 U (IDeg three-times weekly; administered in the evening)  **Titration**  Titrated weekly based on mean pre-breakfast SMBG from previous three days to target of  3.9–<5.0 mmol/L (70–<90 mg/dL) | **Conﬁrmed**  Plasma glucose <3.1 mmol/L  (<56 mg/dL) (irrespective of symptoms) or severe (requiring assistance)  **Nocturnal**  Between 0001 and 0559 h (inclusive) |
| **EDITION trials** | | | | |
| EDITION 1 NCT01499082 Riddle *et al*. Diabetes Care 2014; 37: 2755-62^10^ | Gla-300 vs Gla-100  T2DM requiring BI and prandial insulin | 26 | **Starting dose**  Prior Gla-100 or OD NPH: 1:1  Prior NPH >OD: BI dose reduced 20%  **Titration**  Titrated weekly (and no more than every 3 days) based on mean pre-breakfast SMBG from previous three days to target of 4.4–5.6 mmol/L (80–100 mg/dL)  Prandial insulin titrated on either postmeal  SMBG (target range 6.7–8.9 mmol/L  [121–160 mg/dL) or values before the following meal or at bedtime (5.0–7.2 mmol/L  [90—130 mg/dL) | **Confirmed or severe** Documented symptomatic, asymptomatic (SMBG ≤3.9 mmol/L [≤70 mg/dL] or <3.0 mmol/L  [<54 mg/dL]) or severe events  **Documented symptomatic**  Symptomatic events with SMBG  ≤3.9 mmol/L (≤70 mg/dL) or  <3.0 mmol/L (<54 mg/dL)  **Severe**  Assistance required  **Nocturnal**  Between 0000 and 0559 h (inclusive) |
| EDITION 2 NCT01499095 Yki-Järvinen *et al*. Diabetes Care. 2014; 37: 3235-43^11^ | Gla-300 vs Gla-100  T2DM requiring BI (≥42 units/d) + OADs | 26+26 | **Starting dose**  Prior Gla-100 or OD NPH: 1:1  Prior NPH >OD: BI dose reduced 20%  **Titration**  Titrated weekly based on median pre-breakfast SMBG from previous three days to target of  4.4–5.6 mmol/L (80–100 mg/dL) | **Confirmed or severe** Documented symptomatic, asymptomatic (SMBG ≤3.9 mmol/L [≤70 mg/dL] or <3.0 mmol/L  [<54 mg/dL]) or severe events  **Documented symptomatic**  Symptomatic events with SMBG  ≤3.9 mmol/L (≤70 mg/dL) or  <3.0 mmol/L (<54 mg/dL)  **Severe**  Assistance required  **Nocturnal**  Between 0000 and 0559 h (inclusive) |
| EDITION 3 NCT01676220 Bolli *et al*. Diabetes Obes Metab. 2015; 17: 386-94^12^ | Gla-300 vs Gla-100  Insulin-naïve T2DM on OADs | 26 | **Starting dose**  0.2 U/kg body weight rounded down to the closest whole number divisible by 3  **Titration**  Titrated weekly (and no more than every 3 days) based on mean pre-breakfast SMBG from previous three days to target of 4.4–5.6 mmol/L (80–100 mg/dL) | **Confirmed or severe** Documented symptomatic, asymptomatic (SMBG ≤3.9 mmol/L [≤70 mg/dL] or <3.0 mmol/L  [<54 mg/dL]) or severe events  **Documented symptomatic**  Symptomatic events with SMBG  ≤3.9 mmol/L (≤70 mg/dL) or  <3.0 mmol/L (<54 mg/dL)  **Severe**  Assistance required  **Nocturnal**  Between 0000 and 0559 h (inclusive) |
| EDITION 4 NCT01683266 Home *et al*. Diabetes Care 2015; 38: 2217-25^13^ | Gla-300 vs Gla-100  T1DM requiring BI and prandial insulin | 26 | **Starting dose**  BI: Matched to BI dose at Day −1  **Titration**  BI titrated weekly (and no more than every  3 days) based on mean pre-breakfast SMBG from previous three days to target of  4.4–7.2 mmol/L (80–130 mg/dL)  Prandial insulin: 2-h postprandial PG target  <8.9 mmol/L (<160 mg/dL) | **Confirmed or severe** Documented symptomatic, asymptomatic (SMBG ≤3.9 mmol/L [≤70 mg/dL] or <3.0 mmol/L  [<54 mg/dL]) or severe events  **Documented symptomatic**  Symptomatic events with SMBG  ≤3.9 mmol/L (≤70 mg/dL) or  <3.0 mmol/L (<54 mg/dL)  **Severe**  Assistance required  **Nocturnal**  Between 0000 and 0559 h (inclusive) |
| EDITION JP 1 NCT01689129 Matsuhisa M *et al*. Diabetes Obes Metab 2016; 18: 375-83^14^ | Gla-300 vs Gla-100  T1DM requiring BI and prandial insulin | 26 | **Starting dose**  Prior Gla-100 OD or BID, NPH or IDet OD: 1:1  Prior NPH or IDet >OD: BI dose reduced 20%  **Titration**  BI titrated weekly (and no more than every  3 days) based on mean pre-breakfast SMBG from previous three days to target of  4.4–7.2 mmol/L (80–130 mg/dL)  Prandial insulin: According to approved labelling in Japan | **Confirmed or severe** Documented symptomatic, asymptomatic (SMBG ≤3.9 mmol/L [≤70 mg/dL] or <3.0 mmol/L  [<54 mg/dL]) or severe events  **Documented symptomatic**  Symptomatic events with SMBG  ≤3.9 mmol/L (≤70 mg/dL) or  <3.0 mmol/L (<54 mg/dL)  **Severe**  Assistance required  **Nocturnal**  Between 0000 and 0559 h (inclusive) |
| EDITION JP 2 NCT01689142 Terauchi *et al*. Diabetes Obes Metab. 2016; 18: 366-74^15^ | Gla-300 vs Gla-100  T2DM requiring BI and OAD | 26 | **Starting dose**  Prior Gla-100 OD or BID, NPH or IDet OD: 1:1  Prior NPH >OD: BI dose reduced 20%  **Titration**  BI titrated weekly (and no more than every  3 days) based on mean pre-breakfast SMBG from previous three days to target of  4.4–5.6 mmol/L (80–100 mg/dL) | **Confirmed or severe** Documented symptomatic, asymptomatic (SMBG ≤3.9 mmol/L [≤70 mg/dL] or <3.0 mmol/L  [<54 mg/dL]) or severe events  **Documented symptomatic**  Symptomatic events with SMBG  ≤3.9 mmol/L (≤70 mg/dL) or  <3.0 mmol/L (<54 mg/dL)  **Severe**  Assistance required  **Nocturnal**  Between 0000 and 0559 h (inclusive) |

BI, basal insulin; BID, twice daily; Gla-100, insulin glargine 100 U/mL; Gla-300, insulin glargine 300 U/mL; IDeg-100, insulin degludec 100 U/mL; IDeg-200, insulin degludec 200 U/mL; IDet, insulin detemir; NPH, neutral protamine Hagedorn; OAD, oral antihyperglycaemic drug;
OD, once daily; SMBG, self-monitored blood glucose; T1DM, type 1 diabetes; T2DM, type 2 diabetes.

**References**

[1] Garber AJ, King AB, Del Prato S*, et al.* Insulin degludec, an ultra-longacting basal insulin, versus insulin glargine in basal-bolus treatment with mealtime insulin aspart in type 2 diabetes (BEGIN Basal-Bolus Type 2): a phase 3, randomised, open-label, treat-to-target non-inferiority trial. Lancet. 2012; **379**: 1498-1507

[2] Zinman B, Philis-Tsimikas A, Cariou B*, et al.* Insulin degludec versus insulin glargine in insulin-naive patients with type 2 diabetes: a 1-year, randomized, treat-to-target trial (BEGIN Once Long). Diabetes Care. 2012; **35**: 2464-2471

[3] Gough SC, Bhargava A, Jain R, Mersebach H, Rasmussen S, Bergenstal RM. Low-volume insulin degludec 200 Units/mL once daily improves glycemic control similarly to insulin glargine with a low risk of hypoglycemia in insulin-naive patients with type 2 diabetes: a 26-week, randomized, controlled, multinational, treat-to-target trial: the BEGIN LOW VOLUME trial. Diabetes Care. 2013; **36**: 2536-2542

[4] Onishi Y, Iwamoto Y, Yoo SJ, Clauson P, Tamer SC, Park S. Insulin degludec compared with insulin glargine in insulin‐naïve patients with type 2 diabetes: a 26‐week, randomized, controlled, Pan‐Asian, treat‐to‐target trial. J Diabetes Invest. 2013; **4**: 605-612

[5] Meneghini L, Atkin SL, Gough SCL*, et al.* The efficacy and safety of insulin degludec given in variable once-daily dosing intervals compared with insulin glargine and insulin degludec dosed at the same time daily: a 26-week, randomized, open-label, parallel-group, treat-to-target trial in individuals with type 2 diabetes. Diabetes Care. 2013; **36**: 858-864

[6] Davies MJ, Gross JL, Ono Y*, et al.* Efficacy and safety of insulin degludec given as part of basal–bolus treatment with mealtime insulin aspart in type 1 diabetes: a 26-week randomized, open-label, treat-to-target non-inferiority trial. Diabetes Obes Metab. 2014; **16**: 922-930

[7] Heller S, Buse J, Fisher M*, et al.* Insulin degludec, an ultra-longacting basal insulin, versus insulin glargine in basal-bolus treatment with mealtime insulin aspart in type 1 diabetes (BEGIN Basal-Bolus Type 1): a phase 3, randomised, open-label, treat-to-target non-inferiority trial. Lancet. 2012; **379**: 1489-1497

[8] Mathieu C, Hollander P, Miranda-Palma B*, et al.* Efficacy and safety of insulin degludec in a flexible dosing regimen vs insulin glargine in patients with type 1 diabetes (BEGIN: Flex T1): a 26-week randomized, treat-to-target trial with a 26-week extension. The Journal of clinical endocrinology and metabolism. 2013; **98**: 1154-1162

[9] Zinman B, DeVries JH, Bode B*, et al.* Efficacy and safety of insulin degludec three times a week versus insulin glargine once a day in insulin-naive patients with type 2 diabetes: results of two phase 3, 26 week, randomised, open-label, treat-to-target, non-inferiority trials. The Lancet Diabetes & Endocrinology. 2013; **1**: 123-131

[10] Riddle MC, Bolli GB, Ziemen M, Meuhlen-Bartmer I, Bizet F, Home P. New insulin glargine 300 units/mL versus glargine 100 units/mL in people with type 2 diabetes using basal and mealtime insulin: glucose control and hypoglycemia in a 6-month randomized controlled trial (EDITION 1). Diabetes Care. 2014; **37**: 2755-2762

[11] Yki-Jarvinen H, Bergenstal RM, Ziemen M*, et al.* New insulin glargine 300 units/mL versus glargine 100 units/mL in people with type 2 diabetes using oral agents and basal insulin: glucose control and hypoglycemia in a 6-month randomized controlled trial (EDITION 2). Diabetes Care. 2014; **37**: 3235-3243

[12] Bolli GB, Riddle MC, Bergenstal RM*, et al.* New insulin glargine 300 U/mL compared with glargine 100 U/mL in insulin-naive people with type 2 diabetes on oral glucose-lowering drugs: a randomized controlled trial (EDITION 3). Diabetes Obes Metab. 2015; **17**: 386-394

[13] Home PD, Bergenstal RM, Bolli GB*, et al.* New insulin glargine 300 Units/mL versus glargine 100 Units/mL in people with type 1 diabetes: a randomized, phase 3a, open-label clinical trial (EDITION 4). Diabetes Care. 2015; **38**: 2217-2225

[14] Matsuhisa M, Koyama M, Cheng X*, et al.* New insulin glargine 300 U/ml versus glargine 100 U/ml in Japanese adults with type 1 diabetes using basal and mealtime insulin: glucose control and hypoglycaemia in a randomized controlled trial (EDITION JP 1). Diabetes Obes Metab. 2016; **18**: 375-383

[15] Terauchi Y, Koyama M, Cheng X*, et al.* New insulin glargine 300 U/ml versus glargine 100 U/ml in Japanese people with type 2 diabetes using basal insulin and oral antihyperglycaemic drugs: glucose control and hypoglycaemia in a randomized controlled trial (EDITION JP 2). Diabetes Obes Metab. 2016; **18**: 366-374
